# A study on the rheological properties and modification mechanism of graphene oxide/polyurethane/SBS-modified asphalt

**DOI:** 10.1371/journal.pone.0262467

**Published:** 2022-03-07

**Authors:** Shuai Li, Wenyuan Xu, Fengfa Zhang, He Wu

**Affiliations:** 1 College of Civil Engineering, Northeast Forestry University, Harbin, Heilongjiang Province, China; 2 College of Civil engineering, Heilongjiang Institute of Technology, Harbin, Heilongjiang Province, China; Al Mansour University College-Baghdad-Iraq, IRAQ

## Abstract

To study the effect of graphene oxide (GO) on thermoplastic polyurethane (TPU)/styrene–butadiene–styrene (SBS)-modified asphalt and reveal the modification mechanism, GO/TPU/SBS-modified asphalt was prepared by high-speed shearing Hongjuan et al. (2020). The physical properties of the modified asphalt were measured via a basic index test, and the dynamic rheological behavior of the modified asphalt was characterized by a dynamic shear rheometer (DSR), a bending beam rheometer (BBR), and other technical means. Moreover, double-beam UV-visible (UV-Vis) spectrophotometry and Fourier-transform infrared spectroscopy (FTIR) were conducted to determine the mechanism of asphalt modification from the microscopic perspective. The experimental results reveal that the GO content can improve the basic mechanical properties, high-temperature stability, and low-temperature cracking resistance of TPU/SBS-modified asphalt. When the GO content is 0.5%, the ductility and softening point of the modified asphalt are found to be significantly increased, and the degree of penetration is slightly decreased. Moreover, with the increase of the GO content, the rutting resistance and crack resistance of the asphalt materials are improved. Via the joint action of physical modification and chemical reaction, GO can form a stable structure with asphalt molecules, enhance the stability between asphalt molecules, and increase the colloidal content of macromolecules in the modified asphalt components.

## Introduction

Modified matrix asphalt has been widely used in the pavement and bridge decks of highways, and has achieved satisfactory results. However, with the improvement of the road service level and the extension of the road service time, the effect of asphalt modified with a single modifier under the repeated action of the external environment and driving loads has been unsatisfactory, and the performance of the road surface has not been further improved [1–4]. Therefore, to improve the pavement performance, a variety of asphalt modifiers have been utilized.

In the process of road construction, styrene–butadiene–styrene (SBS)-modified asphalt and polyurethane (PU)-modified asphalt currently play significant roles in improving the pavement performance and extending the pavement life, and are widely used in high-grade asphalt pavement surface layers due to their excellent high- and low-temperature performance and good fatigue resistance [5, 6]. With the development of nanotechnology, nano-modified asphalt has become one of the highlights of agro-scientific research in the field of road materials [7–9]. Nanomaterials are renowned for their excellent specific surface area, small size effect, and macroscopic quantum tunneling effect. The addition of nanomaterials can change the microstructure of asphalt and endow it with excellent thermodynamic and adhesion properties [10–12]. Graphene oxide (GO) has been widely used as a new asphalt modifier due to its unique quasi-two-dimensional layered structure, excellent ability to block oxygen, and good inter-solubility with organic solvents [8, 13, 14]. The use of nano-modified materials can prolong the general service life of modified asphalt pavement infrastructure and reduce the life cycle cost and delay cost during operation. Moreover, the cost of nano-materials has exhibited a downward trend over time, and may be further reduced with the improvement of manufacturing technology [15].

Liu and Gao conducted a dynamic rheological shear test, water stability test, and low-temperature crack resistance test, and found that the addition of the nano-layered silicate material A-PAL improved the high-temperature stability, surface free energy, adhesion, and low-temperature performance of SBS-modified asphalt [6]. Zhou and Zhang prepared GO/SBS-modified asphalt via the high-speed shear method, and found that the introduction of GO can significantly improve the high-temperature stability and dynamic mechanical response of SBS-modified asphalt [2]. Yu et al. used GO/PU nanocomposites for asphalt modification, and found that the modifiers changed the damage properties of the matrix asphalt, which endowed the modified asphalt mixture with better mechanical properties to better resist damage at low temperatures. GO/PU-modified asphalt can improve the properties of the material from the two aspects of alloying and composite. Thus, the addition of a GO/PU modifier can improve the low-temperature crack resistance of asphalt mixture pavement [16].

Previous researchers have found that the nanomaterial GO, either on its own or combined with SBS or PU, can improve the material properties of asphalt. However, it remains unclear whether GO, SBS, and PU materials can improve the performance of multi-composite matrix asphalt. Therefore, it is necessary to further study the pavement performance of GO/PU/SBS-modified asphalt.

## Experimental

### Materials

#### TPU/SBS-modified asphalt

**Fig 1:**
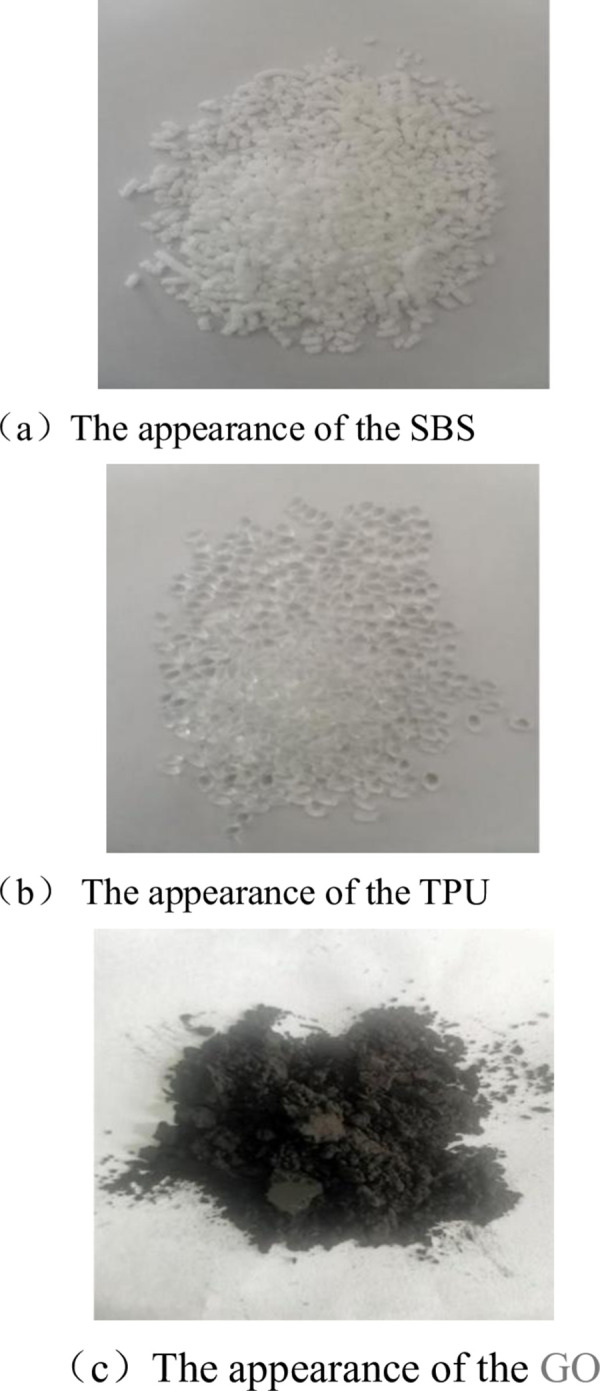
The appearance of the modifiers used in this research. The appearance of the SBS (**a**), TPU (**b**), and GO (**c**).

Thermoplastic polyurethane (TPU)/SBS-modified asphalt was prepared in the laboratory by mixing SBSYH-792E modifier (Sinopec Production) with SK-90 matrix asphalt (Inner Mongolia, China) and TPU elastomer (Germany). The basic specification of SK-90 matric asphalt are shown in Table 1. (The content of SBSYH-792E modifier was 4.5%, and that of TPU was 5%. Based on previous experimental research, it was found that the road performance of TPU/SBS-modified asphalt is the best when the content of the SBSYH-792E modifier is 4.5% and the content of TPU is 5%.) (The pictures of the three modifiers are shown in Fig 1). (The GO specifications used in this study are shown in Table 2.)

**Table 1:** The technical indicators of the SK-90 matrix asphalt used in this research.

	Test results	Technical requirements
Penetration (0.1 mm)	87.7	80–100
Softening point (Global Method) (°C)	47.2	≥45
Ductility (cm)	46.5	≥45
Solubility (%)	102.3	≥99.9
Dynamic viscosity at 60°C (Pa•s)	177.7	≥160

**Table 2:** The technical indicators of the GO used in this research.

	Symbol	Detection of typical values
Place of Origin		Qitaihe City, Heilongjiang Province, China
Appearance		Black powder
Oxygen level	%	44.88
Particle size	D50 (μ)	30.22
Thickness	(μh)	<5
Specific surface area	m^2^·g^-1^	>500

### Graphene oxide (GO)

Table 2.

#### Preparation of GO/TPU/SBS-modified asphalt

To uniformly mix the modifier into the SK-90 matrix asphalt and reduce the precipitation of crystals, the modified asphalt was prepared by first using an asphalt mixer and then using a high-speed shearing machine. First, 500 g SK-90 matrix asphalt was placed in a metal vessel, which was then placed in a constant-temperature oven at 150°C and for 2 h until the asphalt melted. The molten asphalt was then placed in an electric heating plate at 160°C, after which 22.5 g SBSYH-792E modifier and 25 g TPU modifier were successively added, and the asphalt agitator was then turned on. The mixture was stirred at a speed of 300 r/min for 30 min until there were no obvious solid particles. Then, 0.25%, 0.50%, 0.75%, and 1.00% GO (mass proportion) were respectively added to the metal ware of a high-speed shearing machine. The rotor speed was set as 3000 r/min, and high-speed shearing was carried out at 160°C for 45 min. GO/TPU/SBS-modified asphalt was ultimately obtained after full reaction.

### Test methods

#### Basic index test

The penetration degree (25°C), softening point (R&B), and ductility (5°C) of the GO/TPU/SBS-modified asphalt were respectively tested according to the test methods and experimental procedures specified in the Test Rules for Asphalt and Asphalt Mixtures in Highway Engineering (JTG E20-2011) [17].

#### DSR test

**Fig 2:**
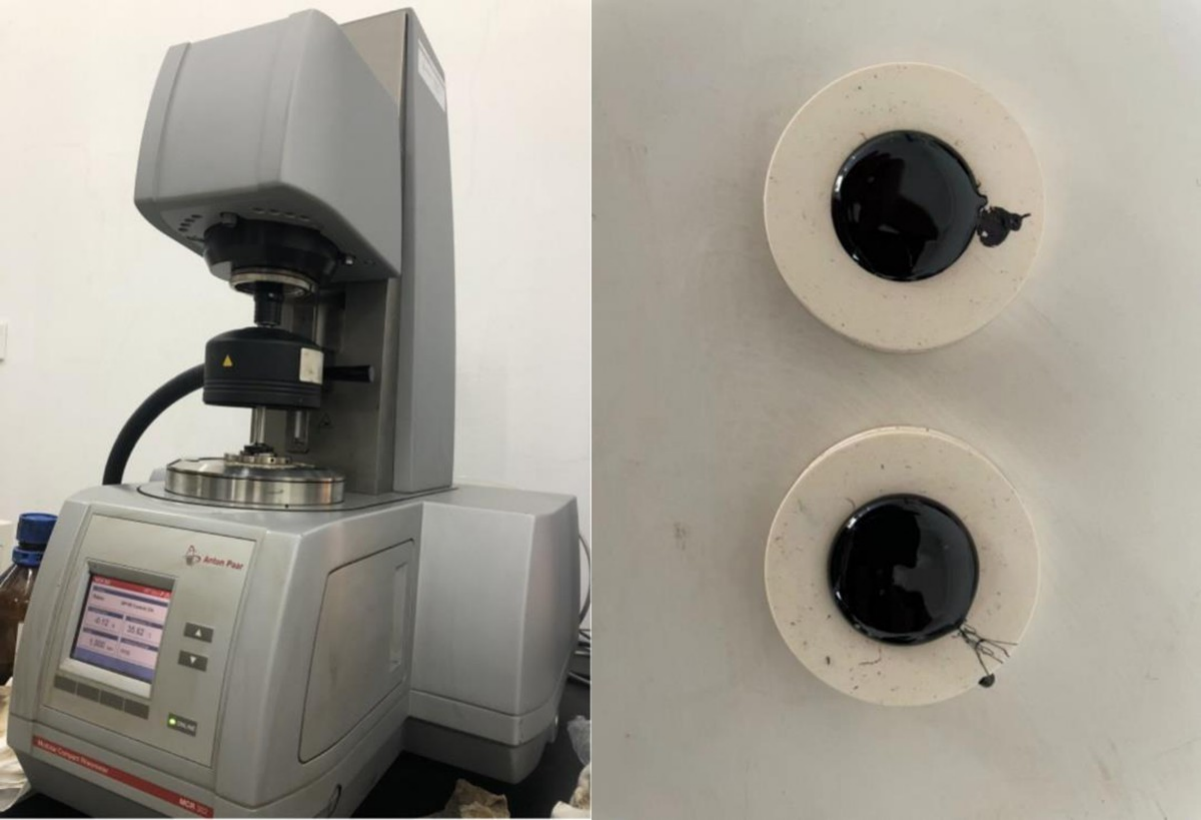
The diagram of the DSR test process.

An advanced dynamic shear rheometer (DSR; Anton Paar MCR model 302e; Fig 2) was used to conduct a temperature scanning experiment to characterize the rheological properties of the GO/TPU/SBS-modified asphalt. A continuous sinusoidal alternating load was applied, and the strain control mode was adopted [18]. The diameter of the parallel plate was 25 mm, and the thickness of the asphalt sample was 1.0 mm. The test temperature was set as 30–85°C, the strain level was 1%, and the rotation frequency was 10 rad/s [14].

#### BBR test

**Fig 3:**
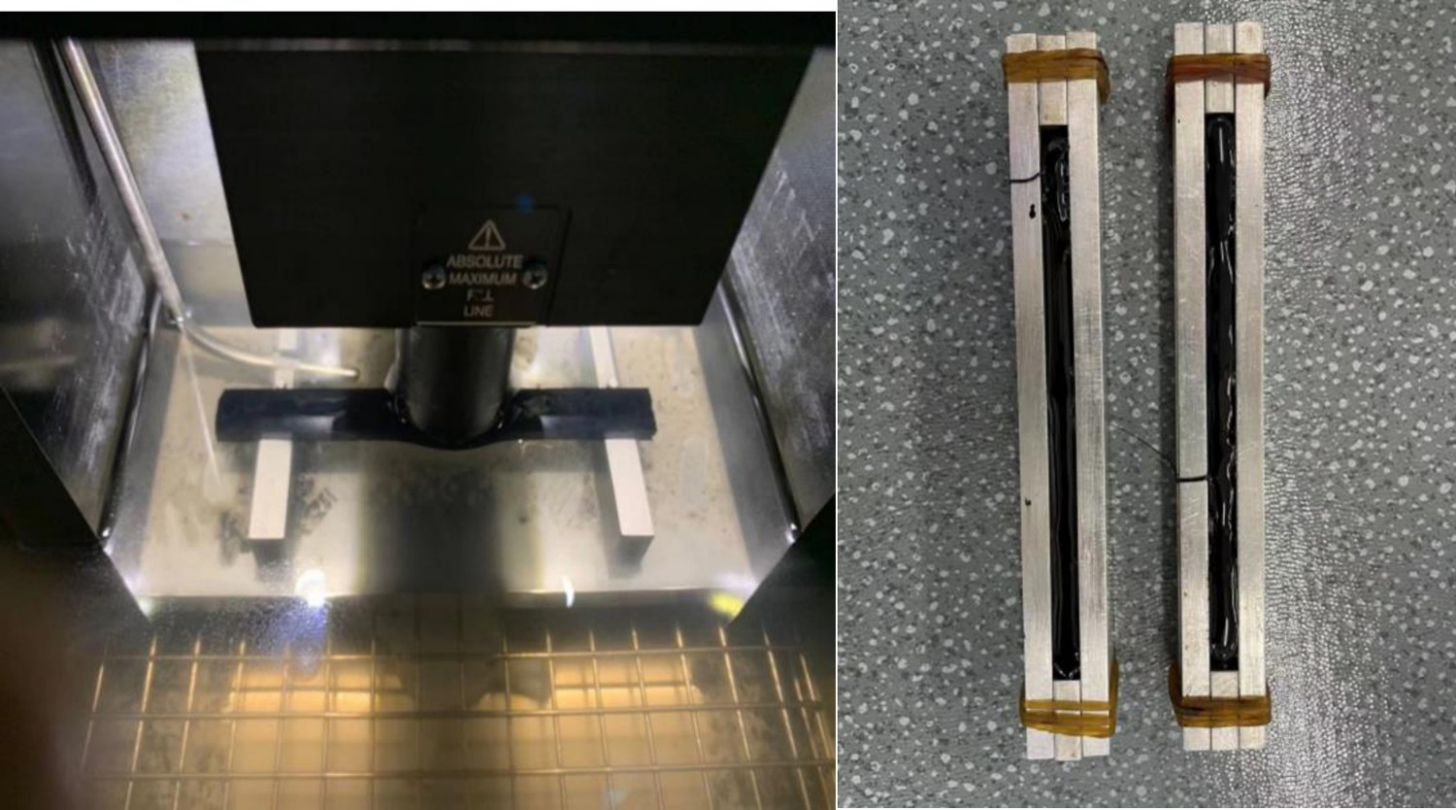
The diagram of the BBR test process.

A bending beam rheometer (BBR; TE-BBR-F, Cannon, USA; Fig 3) test was conducted to evaluate the bending and creep properties of the GO/TPU/SBS-modified asphalt at -16 and -20°C. According to the load and deformation values within 60 s, the creep stiffness *S* and creep velocity *M* of the GO/TPU/SBS-modified asphalt were calculated, according to which the low-temperature crack resistance of the modified asphalt was evaluated [19].

#### Infrared spectrum test

**Fig 4:**
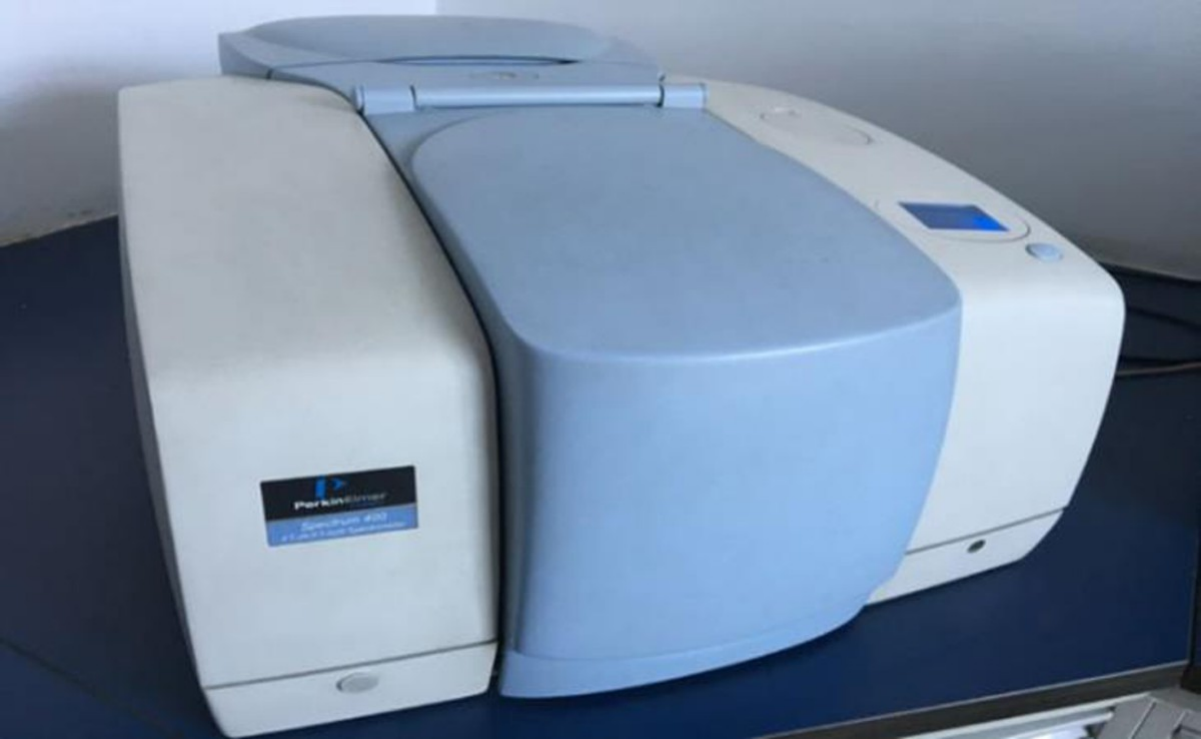
The diagram of the infrared spectrum test process.

The effects of different GO contents on the chemical composition and functional groups of the TPU/SBS-modified asphalt were studied via Fourier-infrared spectroscopy (FTIR; Fig 4). The analysis was performed at the room temperature of 25°C, and the time required for each test (including sample placement and measurement) was less than 5 min. The resolution of the spectrometer was 4 cm^−1^, the number of scans was 32, and the wavenumber test range was 4000 to 400 cm^−1^ [20].

#### UV-Vis test

A UV-visible (UV-Vis) absorption spectrophotometer (TU-1900) was used for the UV-Vis test. The composition, content, and structure of the components of the GO/TPU/SBS-modified asphalt were analyzed, measured, and inferred according to the UV-Vis spectrum and absorption degree generated by the absorption of UV and visible light. The sample asphalt was smeared into a uniform 0.1-mm film on the slide and tested at the room temperature of 25°C.

## Results and discussion

### Basic physical properties of GO/TPU/SBS-modified asphalt

The penetration (25°C), softening point (R&B), and ductility (5°C) tests were carried out in accordance with the relevant requirements and test methods in the Specification.

**Fig 5:**
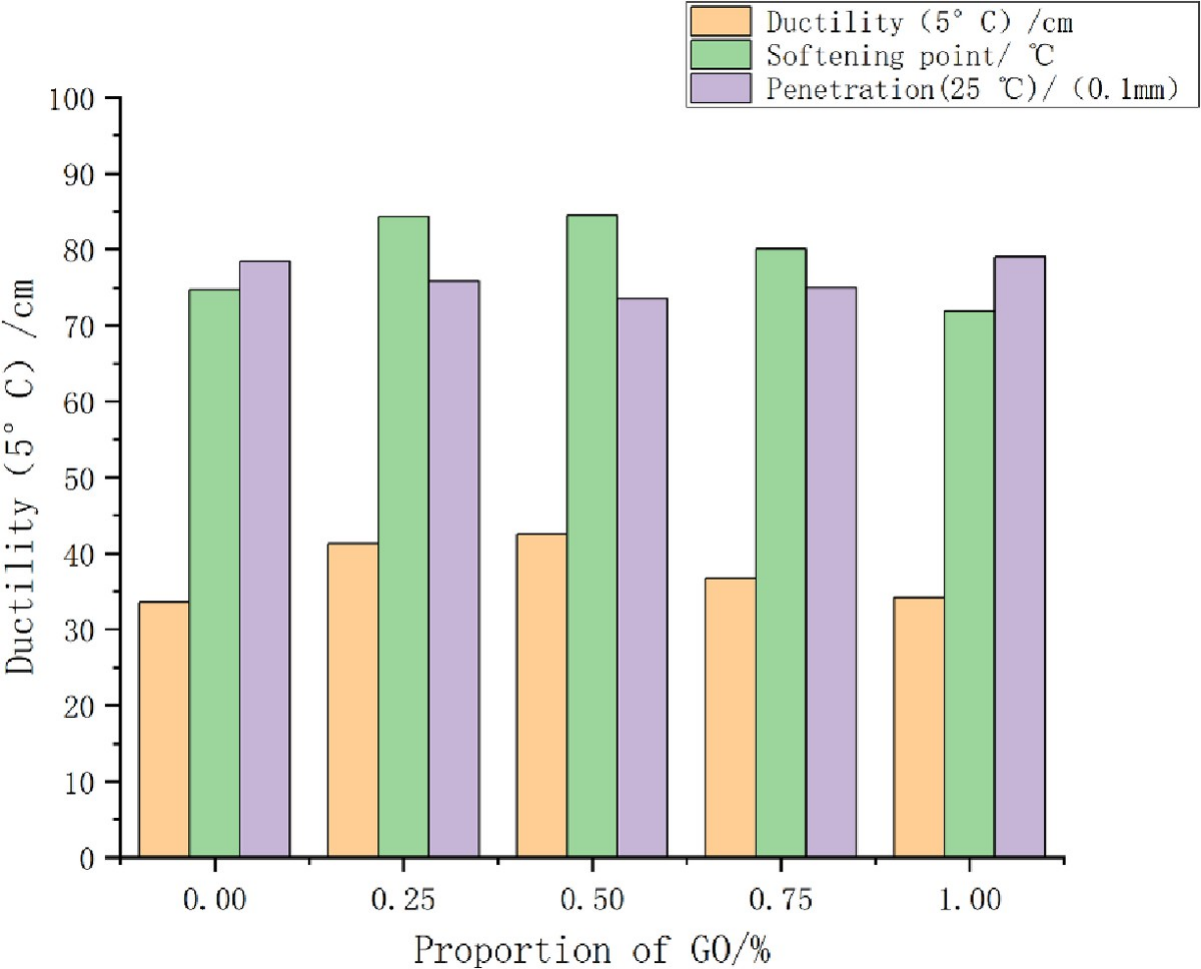
The influence of the GO content on three indicators of the GO/TPU/SBS-modified asphalt.

The influences of different GO contents on the basic indexes of the GO/TPU/SBS-modified asphalt are presented in Fig 5. The degree of penetration of the modified asphalt was found to first decrease and then increase with the increase of the GO content. When the GO content was 0.5%, the degree of penetration reached the minimum value of 73.54 (0.1 mm).

The softening point of the GO/TPU/SBS-modified asphalt exhibited an inverse relationship with the degree of penetration; with the increase of the GO content, the softening point first increased and then decreased. The maximum softening point reached 84.5°C at the GO content of 0.5%.

The variation trend of the ductility of the GO/TPU/SBS-modified asphalt was similar to that of the softening point, namely first increasing and then decreasing. The maximum ductility value reached 42.5 cm at the GO content of 0.5%.

Based on the preceding results, a proper content of GO can improve the basic road performance of TPU/SBS-modified asphalt, and its high-temperature performance, low-temperature performance, and ductility will be improved. However, the excessive addition of GO will form a sliding layer in TPU/SBS-modified asphalt, which will weaken the interaction between asphalt molecules and promote their movement, thereby reducing various performance indicators [21].

### Rheological properties of GO/TPU/SBS-modified asphalt

The complex shear modulus G*, phase angle *δ*, and rutting factor G*/sin*δ* obtained from the DSR test are important parameters for the characterization of the rheological properties of asphalt. The effects of different contents of GO on the rheological behavior of the modified asphalt were studied by temperature scanning (30–85°C). The values of G*/sin*δ* and G*sin*δ* were calculated according to the obtained indexes to evaluate the high-temperature rutting resistance and fatigue cracking resistance of the GO/TPU/SBS-modified asphalt.

**Fig 6:**
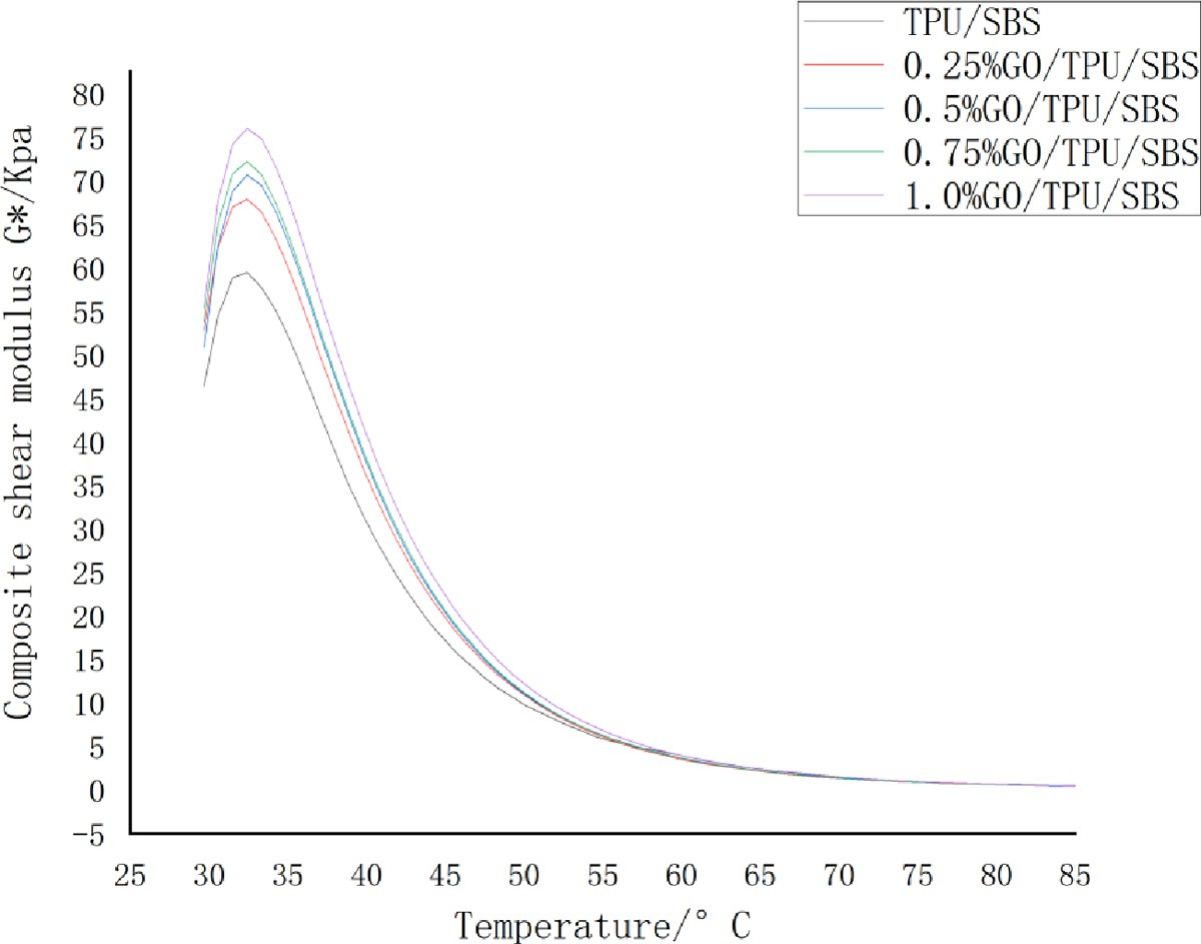
The effect of temperature on the composite shear modulus of the GO/TPU/SBS-modified asphalt.

The complex shear modulus G* is a measure of the total resistance during the repeated shear deformation of the material. The larger the value of G*, the greater the stiffness, the better the high-temperature stability, and the stronger the resistance to flow deformation of asphalt. It can be seen from Fig 6 that with the increase of the temperature from 30 to 85°C, the composite shear moduli of the GO/TPU/SBS-modified asphalt with different GO contents first increased, then decreased sharply, and then gradually decreased. The results indicate that the shear deformation resistance of the asphalt increased from 30 to 35°C, after which it continually decreased from 35 to 85°C. Asphalt exists in a viscoelastic state at low temperatures, and elasticity plays a dominant role. From 30 to 35°C, the sample asphalt changed from a viscoelastic state to a viscous fluid state, and the composite shear modulus underwent a short process of increasing. With the further increase of the temperature, the asphalt sample completely transformed into a viscous fluid state, and the elastic component completely transformed and then disappeared. This led to the decrease of the stiffness of the asphalt and the ability to resist flow deformation, thereby leading to the decrease of the complex shear modulus G*.

Compared with that of the modified asphalt without GO, the G* value of the modified asphalt with GO was obviously improved, as was the resistance to high-temperature shear deformation. This is because, during the preparation process of the modified asphalt, the addition of GO solidifies the component composition of the modified asphalt, forms a more stable structure between the molecular particles, and increases the ability of the modified asphalt to resist high-temperature shear deformation. Therefore, the G* values of the modified asphalt with different contents of GO were higher than those of the modified asphalt without GO.

It can also be seen from the figure that the higher the proportion of GO, the greater the composite shear modulus, but the increase was not very obvious. This is because, with the increase of the GO content, the number of stable-state structures formed between the asphalt molecules increases, and the ability to resist high-temperature shear is enhanced; however, it will gradually approach a saturated state.

**Fig 7:**
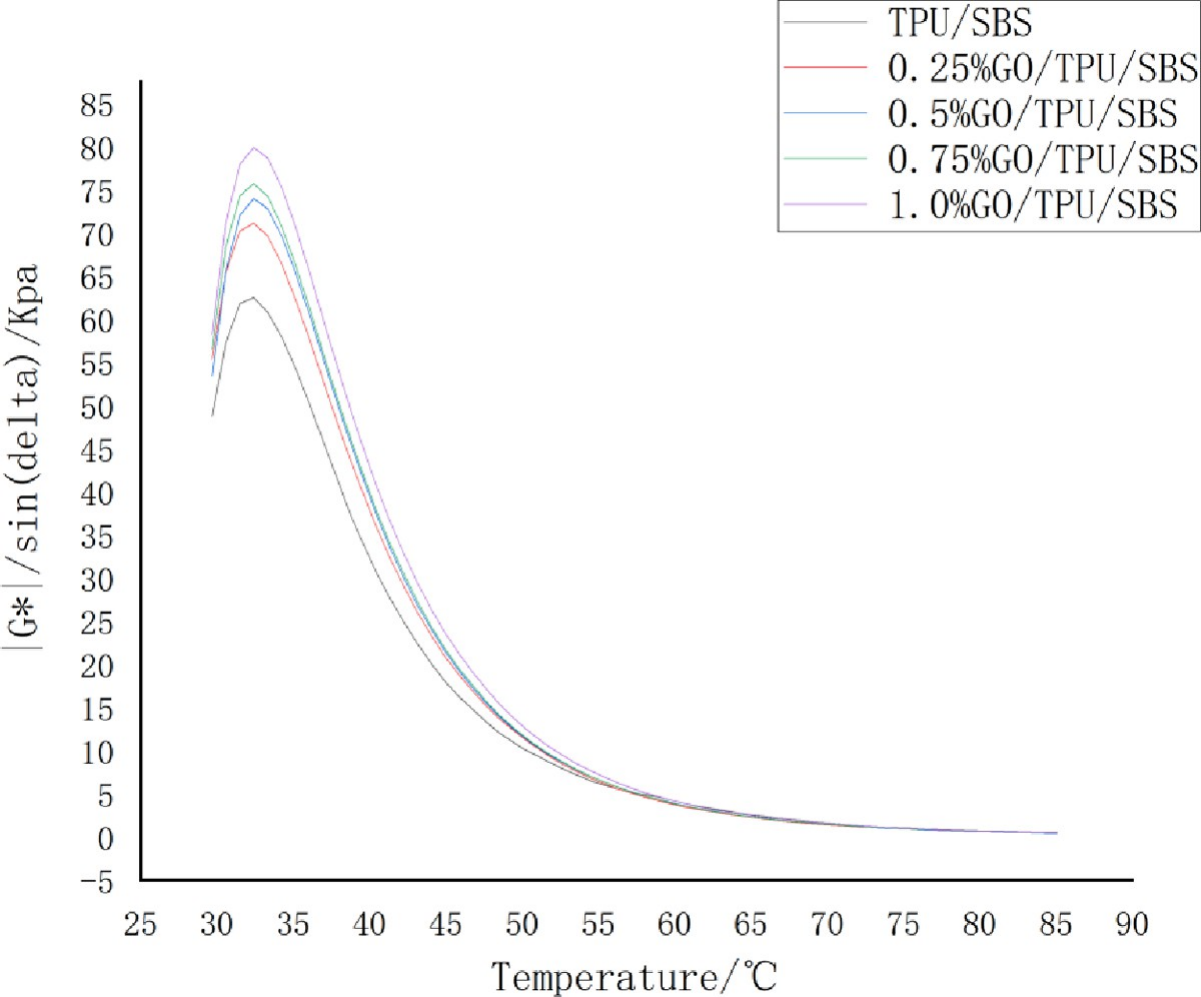
The variation of the rutting factor of GO/TPU/SBS-modified asphalt with the temperature.

The rutting factor G*/sin*δ* is the evaluation index of the high-temperature rutting resistance performance of asphalt. The larger the value of G*/sin*δ*, the smaller the permanent deformation of the asphalt under high-temperature conditions, and the better the rutting resistance effect [22]. As can be seen from Fig 7, with the increase of the temperature, the G*/sin*δ* values of the modified asphalt with different GO contents exhibited a trend of first increasing, then sharply decreasing, and then decreasing gradually, which is similar to the trend of G*. This indicates that the change trend of the rutting factor is greatly affected by G*, and with the increase of the GO content at the same temperature, the G*/sin*δ* value of the modified asphalt will gradually increase. This demonstrates that the addition of GO can improve the plastic deformation resistance and high-temperature shear resistance of asphalt; the higher the GO content, the better the rutting resistance of the modified asphalt at high temperatures.

**Fig 8:**
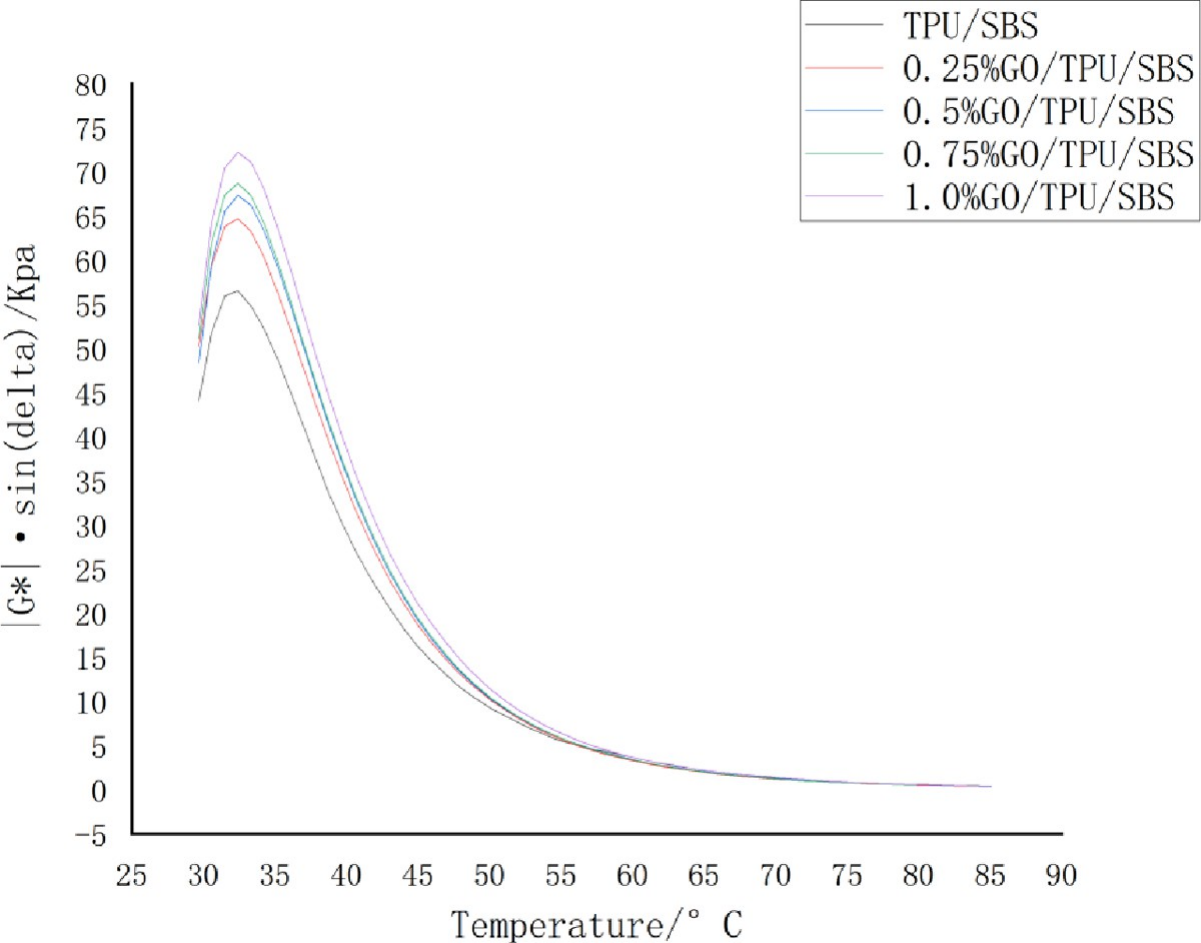
The variation of the G*sinδ values of the GO/TPU/SBS-modified asphalt with the temperature.

G*sin*δ* is often used to evaluate the fatigue cracking resistance of asphalt materials; the lower the G*sin*δ* value, the greater the flexibility and the better the fatigue cracking resistance of asphalt materials. It can be seen from Fig 8 that the G*sin*δ* values all exhibited a trend of first increasing, then decreasing sharply, and then decreasing gradually with the increase of the temperature. Furthermore, with the increase of the temperature, the influence of G*sin*δ* on G* became more obvious. In addition, the higher the proportion of GO, the higher the G*sin*δ* value, and the lower the anti-fatigue cracking ability of asphalt. This may be because more stable structures are formed after combination with the easy flow factors in the asphalt at higher contents of GO; this causes the asphalt to become relatively hard and fragile, thereby reducing its ability to resist fatigue cracking.

The addition of GO was found to reduce the anti-fatigue cracking performance of the modified asphalt, and this effect gradually increases with the increase of the GO content. However, with the increase of the temperature, the G*sin*δ* value of modified asphalt with different GO contents ultimately becomes close to that of modified asphalt without GO, and their anti-fatigue cracking performance is similar.

### Cracking properties of GO/TPU/SBS-modified asphalt at low temperatures

**Fig 9:**
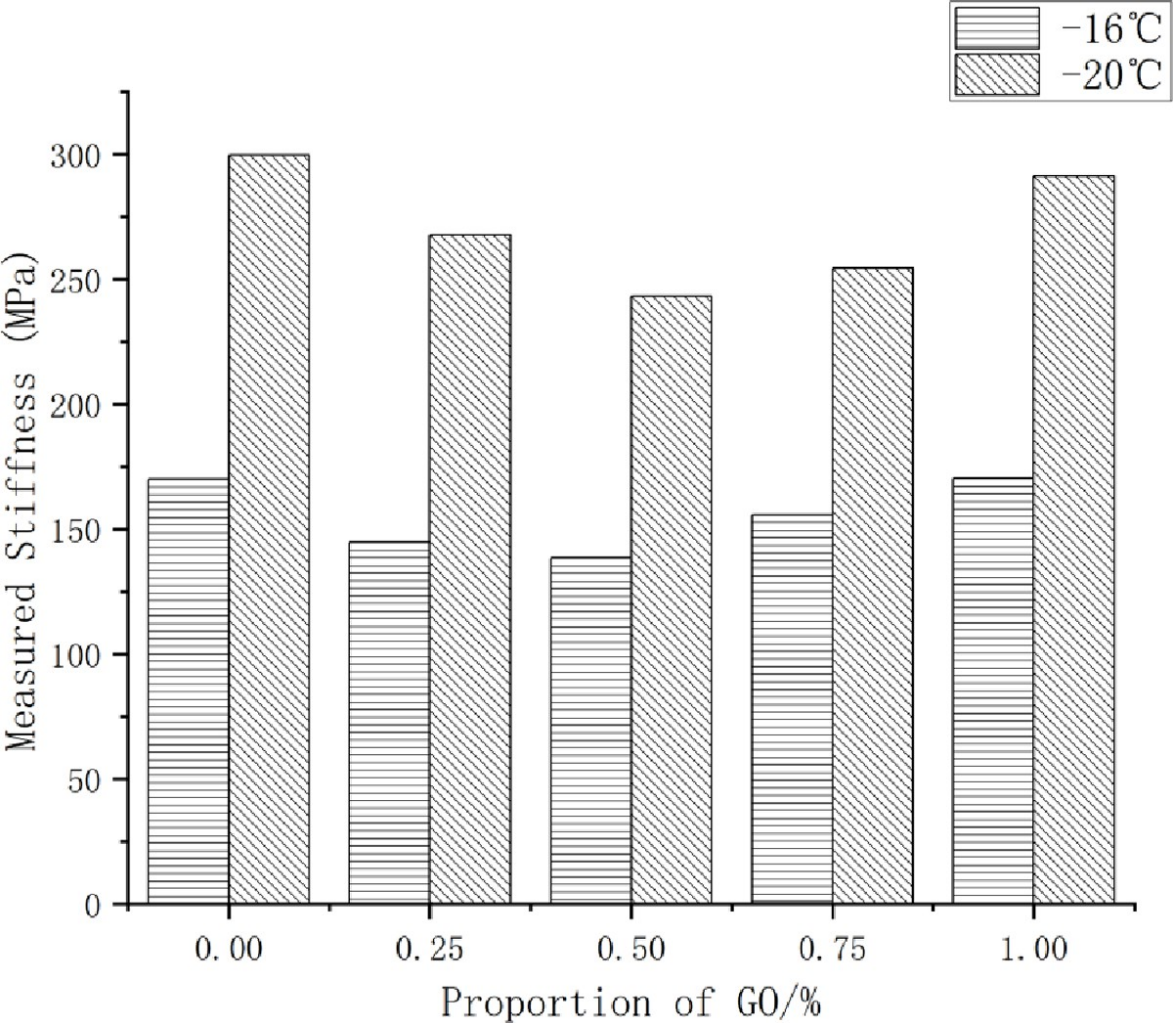
The influences of the GO content on the low-temperature cracking parameters of the GO/TPU/SBS-modified asphalt. (a) Effect on creep stiffness. (b) Effect on creep rate.

The BBR test was conducted to determine the creep stiffness *S* and creep velocity *M* of the modified asphalt samples; *S* represents the ability of asphalt to resist low-temperature deformation, while *M* represents the degree of the stiffness modulus of asphalt changing rapidly with the creep time. The lower the value of *S*, the lower the risk of cracking of asphalt binder at low temperatures. Moreover, the higher the value of *M*, the stronger the stress relaxation ability of asphalt, and the lesser the probability of cracking failure. BBR test results are shown in Fig 9.

The low-temperature crack resistance of the GO/TPU/SBS-modified asphalt was tested via the BBR test. The experimental results reveal that the creep stiffness of all the modified asphalt samples with the addition of GO at -16°C was significantly lower than that at -20°C, while the measured values of creep velocity exhibited an inverse trend, indicating that the low-temperature cracking resistance of the modified asphalt was significantly higher at -16°C than at -20°C.

At -16 and -20°C, the creep stiffness *S* first decreased and then increased with the increase of the GO content, and the lowest value occurred when the GO content was 0.5%. The creep velocity *M* exhibited a trend of increasing first increasing and then decreasing, and the peak value occurred when the GO content was 0.5%. It is evident that the GO/TPU/SBS-modified asphalt exhibited the best low-temperature crack resistance when the GO content was 0.5%.

Compared with those at -16°C, at -20°C, the fluctuation ranges of the creep stiffness *S* and the creep velocity *M* of the modified asphalt with different contents of GO were obviously larger, indicating that with the further decrease of the temperature, the effect of the GO content on the low-temperature performance of modified asphalt will increase.

It can be seen from the test results that an appropriate amount of GO can improve the low-temperature crack resistance, enhance the toughness, and greatly avoid the occurrence of the low-temperature cracking behavior of asphalt. However, the excess incorporation of GO may affect the interaction between asphalt molecules, which will further inhibit the improvement of low-temperature crack resistance and reduce the stress relaxation ability of the modified asphalt.

### Effect of the GO content on asphalt functional groups

FTIR spectra were obtained, and the intensity of the spectral band, including the peak area and peak height, was analyzed. Due to the different GO contents, the atomic groups of each modified asphalt specimen will have different specific vibrations after the molecules are excited, and the characteristic absorption peak appeared in the infrared spectrogram. The quantitative analysis of the material was carried out by analyzing the different absorption peaks.

**Fig 10:**
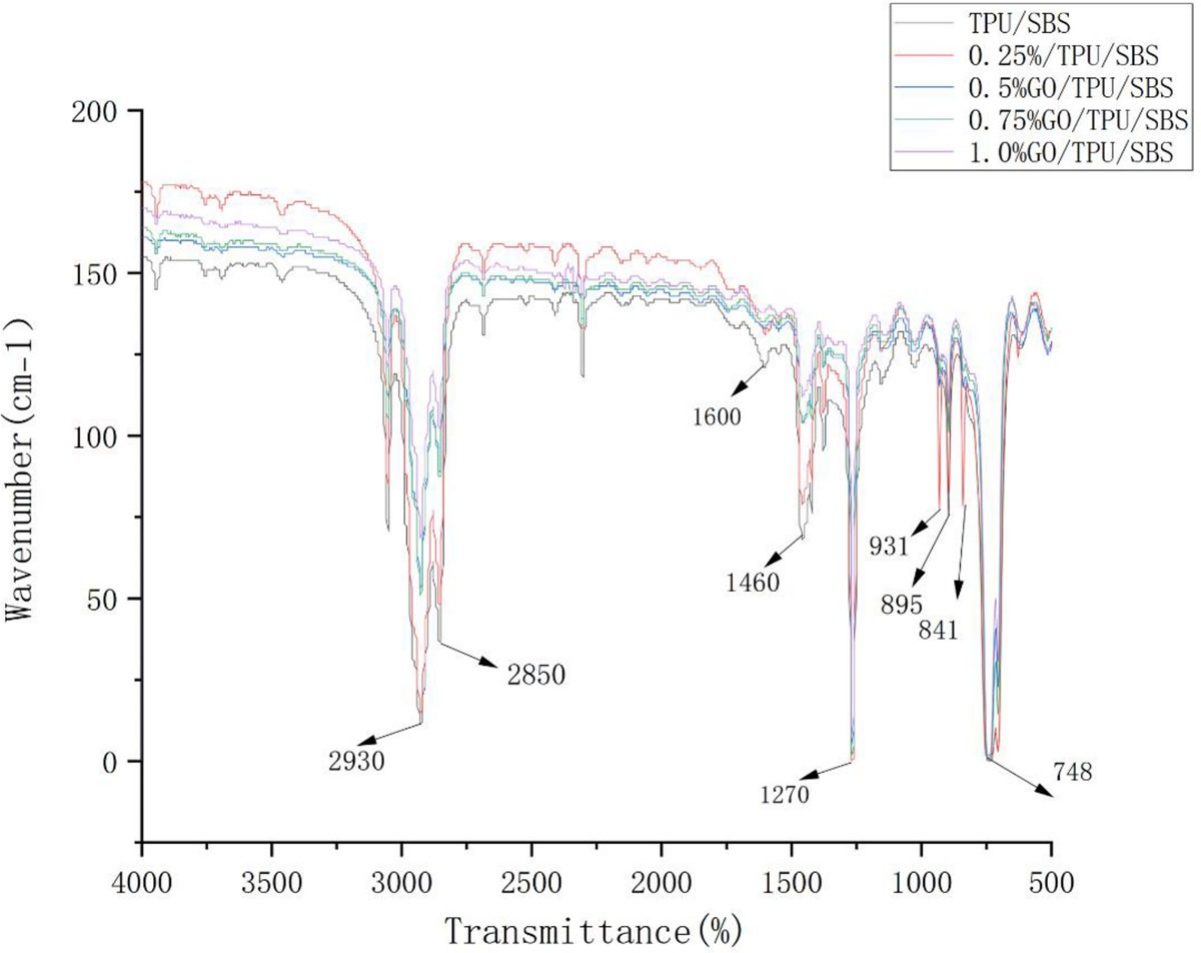
The FTIR test results of modified asphalt with different GO contents.

It can be seen from Fig 10 that the FTIR spectra of the GO/TPU/SBS-modified asphalt exhibited a strong absorption peak in the wavenumber range of 2800–3000 cm^−1^. Generally speaking, the symmetric and asymmetric tensile vibration wavenumbers of -CH- and -CH_2_- in saturated hydrocarbons and their derivatives are less than 3000 cm^-1^. From the absorption peaks at 2930 and 2850 cm^-1^, it can be seen that there existed saturated hydrocarbons and their derivatives in the modified asphalt. Furthermore, with the increase of the GO content in the modified asphalt, the intensity of the absorption peaks at 2930 and 2850 cm^-1^ exhibited a significant weakening phenomenon, indicating that the increase of the GO content reduced the content of saturated hydrocarbons in the modified asphalt.

The vibration of the C = C bond of the conjugated double bond of the benzene ring skeleton of asphalt generated the absorption peak at 1600 cm^-1^. From the absorption peak at 1600 cm^-1^, it can be seen that the infrared spectra of the modified asphalt samples including the addition GO were basically unchanged. However, in the absence of GO, there was an obvious fluctuation phenomenon; this indicates that the addition of GO affected the change of the C = C bonds in the modified asphalt, but the amount of GO had little effect.

The fluctuation of the absorption peak at 1460 cm^-1^ represents the in-plane bending vibration of C-H in the alkane group, and the fluctuation of the absorption peak at 1270 cm^-1^ represents the umbrella vibration of -CH_3_-. Similar to the band of 2800–3000 cm^-1^, it was verified that the increase of the GO content reduced the content of saturated hydrocarbons in the modified asphalt.

The absorption peaks in the region of 900–650 cm^-1^ represent the out-of-plane bending of C-H bonds in aromatic or heteroaromatic groups and the bending vibration of rings. The generation of absorption peaks in this region indicates that the asphalt contained benzene substituents or adjacent hydrogen groups. Furthermore, the peak intensity curves of the five absorption peaks were found to basically coincide in this band. Regardless of whether GO was added or the amount of GO added, the changes of the C-H bonds in the aromatic and heteroaromatic groups were not affected.

It can also be seen from Fig 10 that the fluctuation curves of the infrared spectrum absorption peaks of the modified asphalt with different GO contents were basically the same, and exhibited different peak intensities in the functional group area (4000–1300 cm^-1^) but little difference in the fingerprint area (1300–400 cm^-1^). This indicates that the GO/TPU/SBS-modified asphalt with different GO contents contained basically the same main characteristic functional groups. In the blending reaction of the GO-modified asphalt, both the chemical reaction of chemical bond breaking and physical modification occurred, but physical modification was the main mechanism [23].

### Effect of the GO content on asphalt absorbance

**Fig 11:**
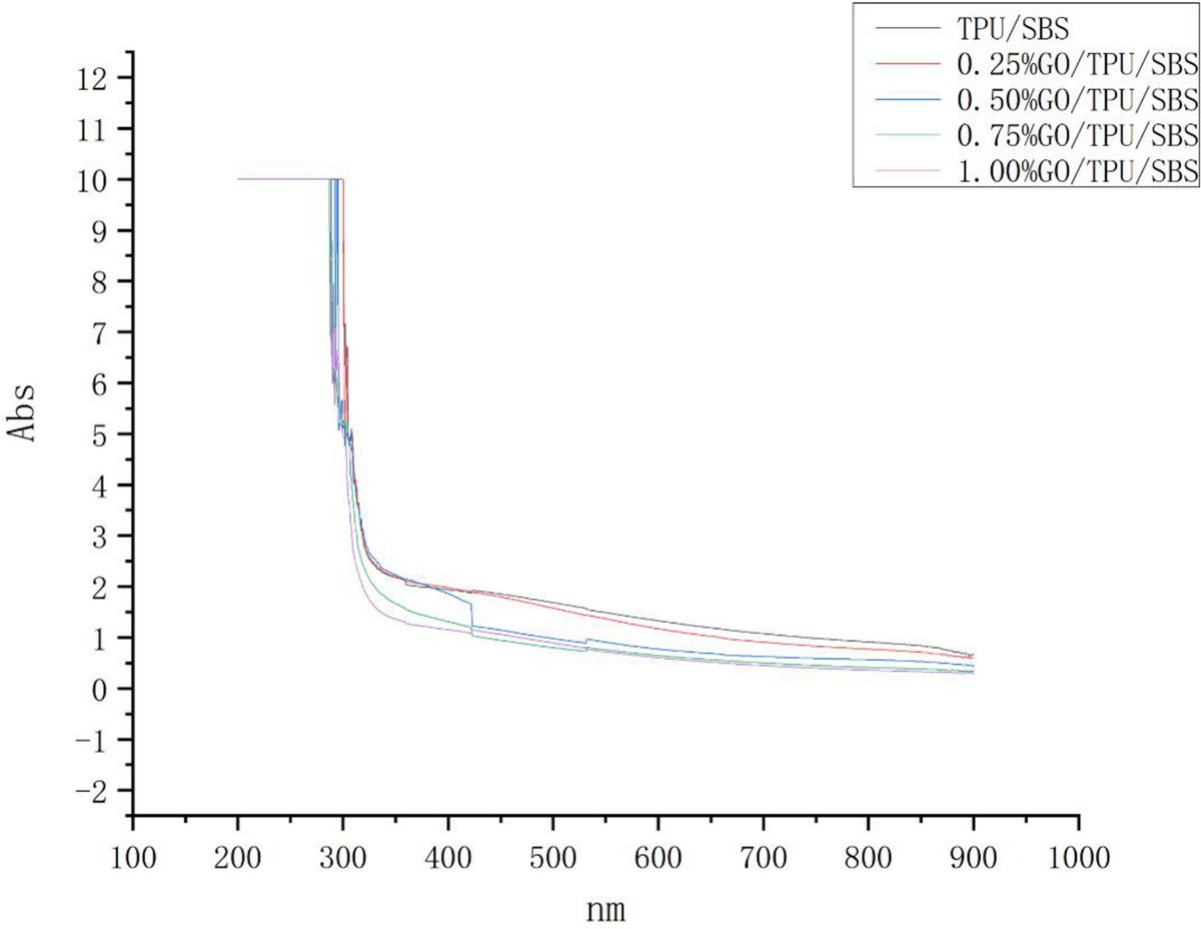
The UV-Vis spectra of the modified asphalt with different GO contents.

The changes in the absorbance of the modified asphalt after the addition of different contents of GO were investigated by a UV-Vis absorption spectrum test, and the particle size and molecular uniformity of the modified asphalt were evaluated. Fig 11 exhibits the UV-Vis spectra of the modified asphalt with different GO contents.

A spectrophotometer was used to measure the absorption spectra of the sample suspensions at 100–1000 nm, among which 100–300 nm was the UV range and 300–1000 nm was the visible range. The absorbance of the samples basically exhibited a trend of translation in the ultraviolet band. In the 250-300-nm band, i.e., at the junction of UV and visible light, there occurred a sharp decrease in absorbance. In the visible light band, the absorbance decreased monotonically with the increase of the wavelength.

GO/TPU/SBS-modified asphalt is a complex dispersive system containing both molecular and coarse dispersive phases. In the UV region, the absorbance of the modified asphalt is mainly caused by the molecular valence electron excitation transition of aromatic hydrocarbons and colloids, but the scattered light is relatively weak. In the visible region, due to the low energy, the probability of molecular valence electron transition is very small. At this time, the absorbance of the sample is primarily due to the light scattering caused by the colloids in the macromolecule. Therefore, the absorbance value was found to decrease with the increase of the wavelength in this region [24].

As can be seen from Fig 11, with the change of the GO content, the absorbance in the UV light region did not change substantially, indicating that the change of the GO content almost did not change the aromatic hydrocarbons, gum, and other components of the modified asphalt. In the visible light region, the absorbance of visible light also decreased with the increase of the GO content, indicating that the increase of the GO content increased the colloid content of the macromolecules in the modified asphalt components.

### Conclusions

Based on the test results and analysis, the conclusions of this study are summarized as follows.

1. The basic performance test results of GO/TPU/SBS-modified asphalt revealed that the addition of GO can improve the softening point (by about 13%) and ductility (by about 26%) of TPU/SBS-modified asphalt, but will reduce the degree of penetration (by about 6.2%).
2. Compared with those of the modified asphalt without GO, the G*/sin*δ* and G*sin*δ* values of the modified asphalt were increased by the addition of GO. With the increase of the GO content, the fatigue cracking resistance of the asphalt decreased, but the high-temperature rutting resistance increased.
3. The low-temperature cracking performance test results indicated that at the same temperature, the addition of GO improved the low-temperature cracking resistance of the asphalt, and the improvement was found to be the most significant when the GO content was 0.5%.
4. The main characteristic absorption peaks of the FTIR spectra of the modified asphalt with different GO contents were found to be similar. The addition of GO mainly caused the physical blending of the modified asphalt, but some chemical reactions still occurred.
5. The UV-Vis spectra of the modified asphalt with different GO contents coincided in the UV region. In the visible region, the absorbance of visible light decreased with the increase of the GO content. The increase of the GO content increased the colloid content of the macromolecules in the modified asphalt components.

The results of this study demonstrate that compared with TPU/SBS-modified asphalt, the addition of GO improves the road performance. It is expected that the current research results will encourage further research on the production of modified asphalt mixtures with GO modifiers. Furthermore, this study has popularization significance for the practical application of high-performance GO/TPU/SBS-modified asphalt.

## Supporting information

S1 Data Basic index test data. (XLS)

S2 Data BBR test data. (XLS)

S3 Data DSR test data. (XLS)

S4 Data Infrared spectrum data. (XLS)

S5 Data UV-Vis data. (XLS)
